# Effects of Cadmium Stress on Bacterial and Fungal Communities in the Whitefly *Bemisia tabaci*

**DOI:** 10.3390/ijms241713588

**Published:** 2023-09-02

**Authors:** Litao Guo, Zhimin Li, Jianping Xu

**Affiliations:** 1 Institute of Bast Fiber Crops, Chinese Academy of Agricultural Sciences, Changsha 410205, China; guolitao@caas.cn (L.G.); lizhimin@caas.cn (Z.L.); 2 Department of Biology, McMaster University, Hamilton, ON L8S 4K1, Canada

**Keywords:** *Bemisia tabaci*, microbial community diversity, cadmium stress, 16S rRNA, ITS

## Abstract

Heavy metal contamination is among the most prominent environmental problems in China, posing serious threats to both ecosystem and human health. Among the diverse heavy metal contaminants, cadmium is the most serious. The whitefly *Bemisia tabaci* is a cosmopolitan pest capable of causing severe damage to a broad range of agricultural crops, especially vegetables. At present, little is known about the effects of cadmium stress on *B. tabaci*, including on its bacterial and fungal communities. In the current study, we investigated the effects of cadmium on bacterial and fungal communities in whiteflies. Meta-barcode sequencing of the 16S rRNA gene revealed that the whitefly bacterial community contained 264 operational taxonomic units (OTUs) belonging to 201 known genera and 245 known species. The top five most frequent bacterial genera were *Rickettsia*, *Rhodococcus*, *Candidatus Portiera*, *Candidatus Hamiltonella*, and *Achromobacter*. Meta-barcode sequencing of the fungal ITS locus revealed that the whitefly fungal community contained 357 OTUs belonging to 187 known genera and 248 known species. The top five most frequent fungal genera were *Wallemia*, *unclassified_f_Dipodascaceae*, *Apiotrichum*, *Penicillium*, and *unclassified_o_Saccharomycetales*. Cadmium exposure reduced the fungal OTU richness but increased the bacterial Shannon and Simpson diversity indices in whiteflies. In addition, upon exposure to cadmium, the microbial community composition in whiteflies changed significantly, with increased prevalence of the bacterial genera *Rhodococcus* and *Exiguobacterium* and fungal genus *Wallemia*. Our results indicate that the whitefly microbiota likely contributed to their adaptation and resistance to cadmium and suggested that whiteflies may contain microbes that could help remediate cadmium contamination in natural environments and agricultural fields.

## 1. Introduction

Contamination by heavy metals is a common environmental problem and has been attracting increasing attention around the world [1]. In China, heavy metal contamination is among the most prominent environmental concerns [2,3]. In 2014, the China National General Survey for Soil Contamination estimated that 16.1% of the total land surface soil in China had excessive concentrations of heavy metals, with cadmium as the most common heavy metal contaminant [4].

Heavy metals can become hazardous to humans and the environment and can be accumulated through the food chain. Previous studies have shown that heavy metals can also be toxic to plants and invertebrates, including phytophagous insects, threatening the health of organisms across all trophic levels [5,6]. As an important component of the food web in ecosystems, plant-feeding insects are important media for heavy metal dispersal and accumulation in the environment. Indeed, many studies have shown that the accumulation of heavy metals can cause a variety of adverse effects on insects [7]. Interestingly, low levels of some heavy metals can have stimulatory effects, while high levels show almost universal inhibitory effects on biological processes [7]. Specifically, high concentrations of heavy metals can negatively impact the survival, longevity, growth, development, egg hatching, and fecundity of phytophagous insects. In addition, the presence of heavy metals can negatively influence insects’ biochemical, physiological, and behavioral responses to other stressors [1,7]. For example, the expression of metallothionein genes (*OcMT1* and *OcMT2*) and heat shock protein genes (*OcHSPs*) showed obvious changes after cadmium exposure in *Oxya chinensis*, influencing their response to heat stress [8,9]. In cadmium-stressed beet armyworms, the expression of the vitellogenin gene (*Vg*) was significantly down-regulated, delaying egg development [10]. Cadmium stress also down-regulated the expression of odor-binding protein-encoding genes (*OBP7* and *OBP15*), disrupting chemosensory response and olfactory signal transduction in fire ants [11,12]. However, there is very limited knowledge about the effects of heavy metals on microorganisms in insects.

The microbiota has shown to play an important role in nutrient acquisition for host insects and in influencing insect physiology and behavior. As a result, the insect microbiota has drawn increasing attention from the scientific community [13]. The insect microbiota can detoxify toxic secondary metabolites and xenobiotics, for example, nicotine, terpenes, and insecticides [14]. The microbiota can also generate and transmit signals among hosts; protect hosts against pathogens; and modify host immunity and behavior [15]. In the honeybee (*Apis mellifera*), olfactory learning and memory abilities require a healthy gut microbiota, with a key function in regulating tryptophan metabolism [16]. Specifically, a host-specific *Lactobacillus* strain could convert tryptophan to indole derivatives which then could activate the host aryl hydrocarbon receptor in honeybees [16].

The whitefly *Bemisia tabaci* (Gennadius) (Hemiptera: Aleyrodidae) is a broadly distributed and extremely harmful agricultural pest [17]. It causes damage to more than 600 species of plants, including many cash crops [18]. Taxonomically, *B. tabaci* represents a large species complex, comprising more than 35 sibling species and varieties with different biological and genetic traits [19]. Among them, *B. tabaci* MED (Mediterranean) and *B. tabaci* MEAM1 (Middle East-Asia Minor 1) are two of the most invasive and harmful to agriculture in many parts of the world [19]. They can damage plants both directly and indirectly. Directly, whiteflies can pierce plant tissue, suck phloem sap, and secret honeydew. Indirectly, they can transmit more than 300 kinds of plant viruses [20]. Interestingly, *B. tabaci* was found to contain one protosymbiotic bacterium of the genus *Candidatus_Portiera* and many secondary symbiotic bacteria in genera such as *Rickettsia*, *Candidatus_Hamiltonella*, *Wolbachia*, *Arsenophonus*, and *Cardinium* [21,22]. *Candidatus_Portiera*, *Candidatus_Hamiltonella*, and *Arsenophonus* are always located in bacteriocytes, and they supplement their host’s diet by providing a diversity of nutrients [23]. Bacteria in the genera *Cardinium*, *Wolbachia*, and *Rickettsia* are located in different tissues in whiteflies [24].

Thus far, none of the studies on whitefly microbiomes have investigated the potential effects of heavy metal exposure on the microbial community structure in whiteflies. Due to the high prevalence of both heavy metal contamination and whiteflies in agricultural fields, it is important to understand how heavy metal exposure can potentially influence the whitefly microbiome. In this study, we analyzed the influence of one of the most common heavy metal contaminants, cadmium, on the microbiota in *B. tabaci*. Specifically, we established three treatment groups for whiteflies: (i) group #1 receiving no cadmium (CK), (ii) group #2 receiving a low dose (LD) of cadmium, and (iii) group #3 receiving a high dose (HD) of cadmium. We then investigated their bacterial and fungal communities using high-throughput 16S rDNA and ITS meta-barcode sequencing, respectively. The obtained data were analyzed and compared among treatments using principal co-ordinate analyses (PCoAs) and several microbial diversity indices (e.g., Sobs, Shannon, Simpson, ACE, and Chao1). These diversity indices emphasize different components of microbial diversity in each sample and are commonly used together to provide a more comprehensive understanding of microbial communities than using only one or two indices [25]. Finally, we predicted and compared the functions of microbial communities observed in the three treatment groups. Our data provide insights into the potential mechanism of interactions between whiteflies and their microbiota in the presence of cadmium. This research contributes to understanding the role of the microbiota in the whitefly’s adaptation to heavy metals.

## 2. Results

### 2.1. Death Rate of B. tabaci after Exposure to Different Cadmium Concentrations

We observed that as cadmium concentration increased, the death rate of *B. tabaci* also increased (Table 1). The death rates of *B. tabaci* after 48 h of cadmium exposure were 74.6%, 65%, 41.35%, 26.05%, and 14.93% for cadmium concentrations of 120, 60, 30, 15, 5, and 0 µM, respectively (Table 1). The regression equation of cadmium toxicity for *B. tabaci* was Y = −2.1 + 1.33X, with an estimated LC50 value of 37.93 μmol/L and an estimated LC10 value of 4.12 μmol/L. The estimated 95% confidence interval for LC50 was 35.11–40.97 μmol/L. The coefficient of correlation (R) between cadmium concentration and the whitefly death rate was 0.98 (*p* < 0.05).

### 2.2. Effects of Exposure to Low and High Concentrations of Cadmium on Bacterial Communities in Whiteflies

The bacterial community compositions in whiteflies exposed to the CK (no cadmium in diet), LD (0.186 mg of cadmium per kg of diet food), and HD (1.71 mg of cadmium per kg of diet food) treatments were investigated by 16S rRNA amplicon sequencing. Our data contained a total of 457,045 reads with an average read length of 407 bp. The obtained sequences were clustered into 264 bacterial OTUs. These bacterial OTUs belonged to 21 phyla, 201 genera, and 245 known species (Tables S1 and S3). Rarefaction curves from the original sequence data suggested that all 12 samples approached saturation, indicating that the sequencing depth for each sample was sufficient to uncover most bacterial diversity within individual samples (Figure S1A).

**Figure 1:**
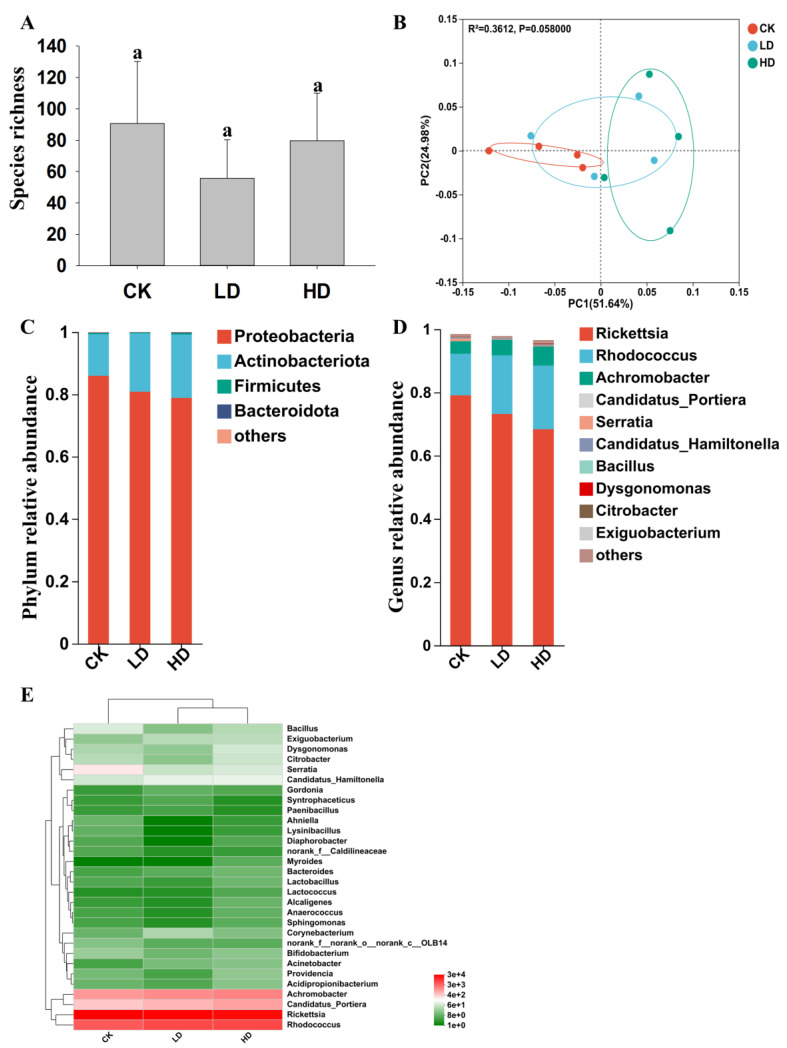
Comparison of bacterial communities in *B. tabaci* with (LD and HD) or without (CK) exposure to cadmium. (**A**) Species richness. Vertical bars represent standard deviations (*n* = 4). Means with the same letter are not significantly different. (**B**) Principal coordinate analysis (PCoA) of bacterial communities based on the pairwise sample Bray–Curtis distances. The significant value of beta diversity was obtained by Adonis analysis with 999 permutations, *p* < 0.05. (**C**,**D**) Composition of bacterial communities among the three treatments based on their relative abundances of bacterial phyla (**C**) and genera (**D**). (**E**) Heatmap of bacterial communities in whiteflies treated with different concentrations of cadmium. Only the top 30 most abundant genera are shown in (**E**). Hierarchical cluster analysis based on composition of genera was performed using the Bray–Curtis distance with average method.

Quantitative differences were found among the three treatment groups in the mean numbers of OTUs (Sobs) within each sample (Table 2 and Table S3). However, none of the pairwise comparisons among the three treatment groups showed that their differences in Sobs were statistically significant (Figure 1A, Table 2 and Table S3). Similarly, no statistically significant difference was found among the three treatment groups for the ACE and Chao1 indices for their bacterial communities. However, the HD treatment group showed significantly higher Shannon diversity than the CK group (Table 2). In addition, both the LD and HD treatments had a higher Simpson’s diversity index than the CK treatment, consistent with the greater overall bacterial diversity in the LD and HD groups than in the CK group.

The bacterial OTUs among the 12 samples were further analyzed through a PCoA based on pairwise Bray–Curtis distances of bacterial OTU compositions. The PCoA showed consistent differences in bacterial OTU compositions among the three treatment groups, with the CK samples clearly separated from HD samples and with the LD samples in between the CK and HD samples (ADONIS test, *p* < 0.05) (Figure 1B). At the phylum level, OTUs belonging to Proteobacteria, Actinobacteriota, Firmicutes, and Bacteroidota dominated all three treatments (Figure 1C). At the genus level, the OTUs of the CK group mainly belonged to the genera *Rickettsia* (80.67%), *Rhodococcus* (13.54%), *Achromobacter* (4.04%), *Candidatus_Portiera* (1.50%), and *Candidatus_Hamiltonella* (0.19%); the OTUs of the LD group were mainly in the genera *Rickettsia* (73.57%), *Rhodococcus* (18.84%), *Achromobacter* (5.00%), *Candidatus_Portiera* (2.18%), and *Candidatus_Hamiltonella* (0.30%); and the OTUs of the HD group mainly belonged to the genera *Rickettsia* (69.62%), *Rhodococcus* (20.21%), *Achromobacter* (6.06%), *Candidatus_Portiera* (3.62%), and *Candidatus_Hamiltonella* (0.36%) (Figure 1D). Figure 1E shows the distributions of the 30 most abundant genera among the three treatment groups. While the genera *Rickettsia*, *Rhodococcus*, *Achromobacter*, and *Candidatus_Portiera* dominated all three treatment groups, their relative frequencies among the three groups differed (Figure 1D,E).

### 2.3. Effective Number of Sequences in Selected Bacterial Genera and OTU Distributions

**Figure 2:**
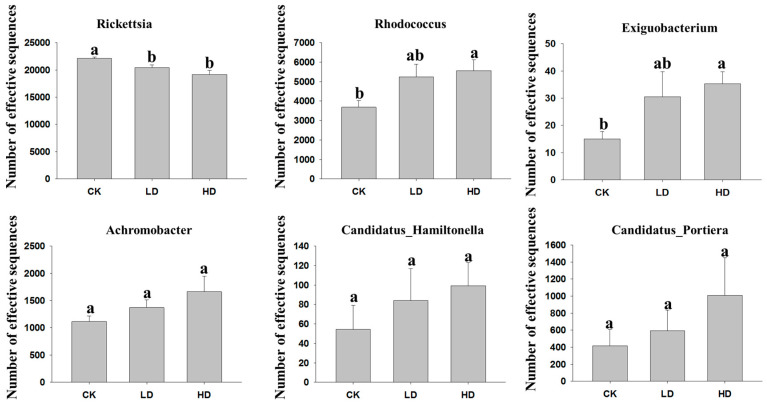
Number of effective sequences in six dominant bacterial genera in CK, LD, and HD treatment groups. Vertical bars represent standard error (*n* = 4). Different letters above columns indicate significant differences (one-way ANOVA, LSD test, *p* < 0.05) in their mean values.

To further compare the effects of cadmium on bacterial diversity in *B. tabaci*, we selected the dominant genera in the total sample and compared their effective number of sequences among the CK, LD, and HD treatments. The results revealed significant differences among the CK, LD, and HD groups in the effective number of sequences in the genera *Rickettsia*, *Rhodococcus*, and/or *Exiguobacterium* (Figure 2). Specifically, the CK group had a more effective number of sequences in the genus *Rickettsia* than the LD and HD groups. In contrast, the LD and HD groups had more effective numbers of sequences in the genera *Rhodococcus* and *Exiguobacterium* than the CK group. Overall, the effective number of sequences in these three genera in the LD group were in between the CK and the HD groups (Figure 2). A similar pattern was observed for the other three common genera *Achromobacter*, *Candidatus_Portiera*, and *Candidatus_Hamiltonella*, but those differences were statistically insignificant (Figure 2). At the OTU level, there were 100 shared OTUs among the three treatments, and the number of unique OTUs of CK, LD, and HD were 26, 17, and 28, respectively (Figure S2). The detailed similarities and overlaps of bacteria at three taxonomic levels (phylum, genus, and OTU) among the three treatments are shown in Figure S2.

### 2.4. Functional Predictions of Bacterial Communities

**Figure 3:**
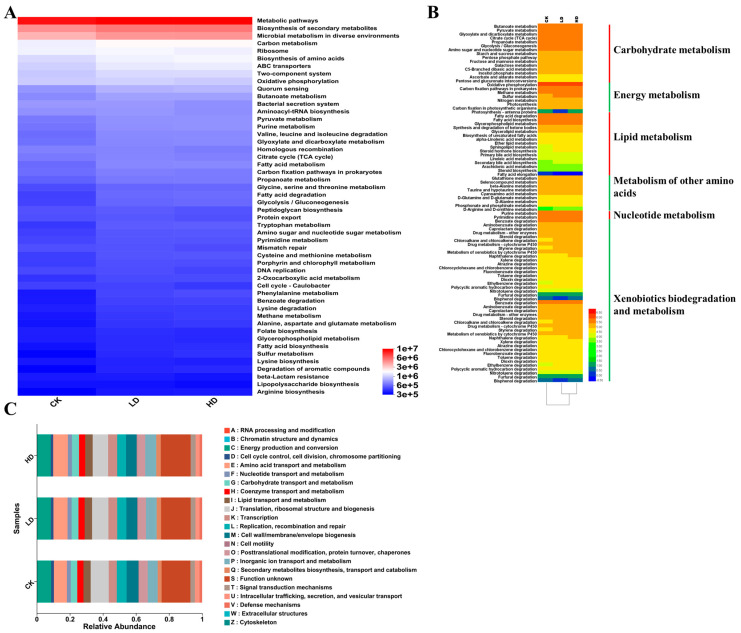
Predicted by PICRUSt2 of bacterial functional groups in *B. tabaci* under different concentrations of cadmium. (**A**) Heatmap of predicted KEGG pathways. (**B**) Heatmap of predicted metabolism pathways. The inferred metabolic pathways included “xenobiotics biodegradation and metabolism”, “nucleotide metabolism”, “metabolism of other amino acids”, “lipid metabolism”, “energy metabolism”, and “carbohydrate metabolism”. (**C**) Relative abundances of predicted COG functions for bacteria in *B. tabaci* among the three treatment groups.

The potential bacterial metabolic pathways (KEGG) and functions (for example, COG) in each sample were analyzed to better understand the putative roles of the bacterial community in *B. tabaci*. The relevant results are shown in Figure 3. The KEGG pathway predictions revealed that in all three treatment groups, the relative abundance of “metabolic pathways” was the highest, followed by “biosynthesis of secondary metabolites” and “microbial metabolism in diverse environments” (Figure 3A). In addition, compared to the CK treatment, cadmium treatments increased the relative abundances of these three KEGG pathways, with the HD treatment showing greater increases than the LD treatment. Similarly, the relative abundances of the pathways “energy metabolism”, “lipid metabolism”, “metabolism of other amino acids”, and “xenobiotics biodegradation and metabolism” also increased in the two cadmium-treated groups compared to the CK (Figure 3B). The results of the COG functional predictions are displayed in Figure 3C. Here, the “function unknown” category was the most abundant in all samples, followed by “translation, ribosomal structure and biogenesis”, and “energy production and conversion”. However, there was a limited difference in the relative abundances of these predicted COG pathways among the three treatment groups (Figure 3C).

### 2.5. Fungal Community Compositions

The fungal community compositions in whiteflies under the CK, LD, and HD treatments were investigated by ITS amplicon sequencing. In total, we obtained 657,407 ITS sequences, and the average length of the ITS sequences was 189 bp. The obtained ITS sequences were clustered into 357 fungal OTUs. These fungal OTUs belonged to 11 phyla, 187 known genera, and 248 known species (Tables S2 and S3). Rarefaction curves from the original sequencing data suggested that all 12 samples reached OTU saturation, indicating that our sequencing depth was sufficient to uncover the total fungal diversity within each sample (Figure S1B).

**Figure 4:**
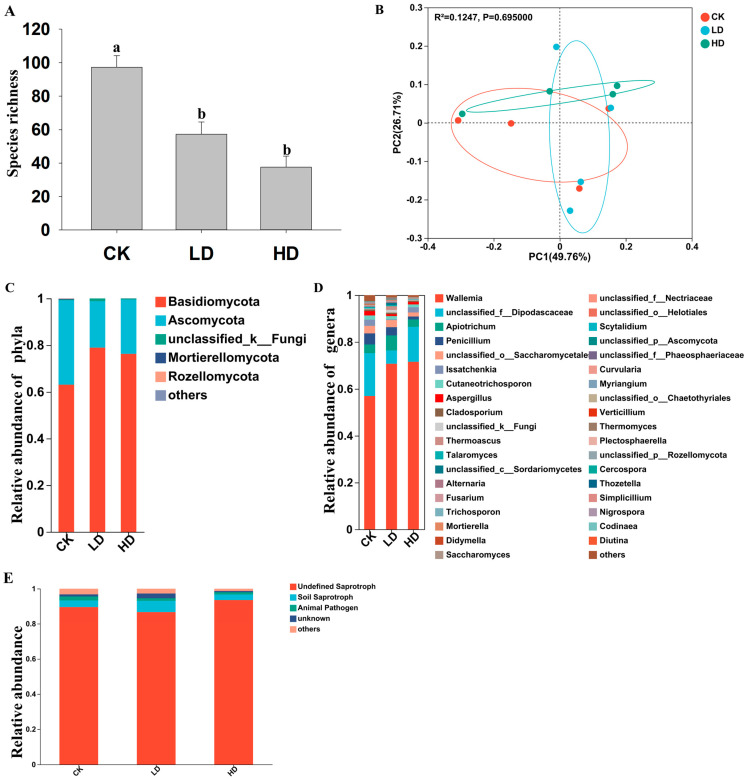
Fungal community compositions and functional predictions in *B. tabaci* treated with different concentrations of cadmium. (**A**) Species richness, vertical bars represent standard error (*n* = 4). Means with the same letter are not significantly different. (**B**) Principal coordinate analysis (PCoA) of fungal communities based on the Bray–Curtis distance. The significance of beta diversities was obtained by Adonis analysis with 999 permutations, *p* < 0.05. (**C**,**D**) Composition of fungal communities in whiteflies treated with different concentrations of cadmium at the phyla level (**C**) and the genus level (**D**). (**E**) Predicted fungal functional groups in *B. tabaci* samples treated with different concentrations of cadmium as predicted by FUNGuild.

Quantitative differences were found among the three treatment groups in their mean numbers of fungal OTUs (Sobs), with the CK group having a greater number of fungal OTUs than both the LD and HD groups (Figure 4A, Table 2 and Table S3). Similarly, the CK group had a higher ACE index and Chao1 index than both the LD and HD groups (Table 2). In addition, the LD treatment group had a higher ACE index than the HD treatment. However, though the CK treatment group had an overall higher Shannon index and Simpson’s index of diversity (1-D) than the LD and HD treatment samples, those differences were statistically insignificant (Table 2).

Principal co-ordinate analysis (PCoA) based on Bray–Curtis distances between pairs of samples showed that the two main principal co-ordinates explained 76% of the total fungal diversity variation among the 12 samples (ADONIS test with 999 permutations, *p* < 0.05) (Figure 4B). However, different from that for the bacterial community, the PCoA failed to separate fungal communities of the 12 samples into three distinct groups based on their cadmium treatments (Figure 4B). Specifically, there were broad variations among replicates within each of the three treatment groups in their OTU distributions, leading to significant overlaps among the treatment groups. At the phylum level, two phyla, Basidiomycota and Ascomycota, dominated the fungal community in all 12 samples in the three treatment groups (Figure 4C). Among the three treatment groups, the CK group had relatively more OTUs belonging to Ascomycota than the LD and HD groups. In contrast, the LD and HD groups had relatively more OTUs belonging to Basidiomycota than the CK group (Figure 4C). At the genus level, in the CK group, the following five genera were the most abundant: *Wallemia* (56.97%), *unclassified_f_Dipodascaceae* (18.23%), *Penicillium* (4.74%), *Apiotrichum* (3.69%), and *unclassified_o_Saccharomycetales* (3.22%). In the LD treatment group, the top five dominant genera were *Wallemia* (70.66%), *Apiotrichum* (6.42%), *unclassified_f_Dipodascaceae* (5.68%), *Penicillium* (3.54%), and *unclassified_o_Saccharomycetales* (3.01%). In the HD samples, the top five most abundant genera were *Wallemia* (71.51%), *unclassified_f_Dipodascaceae* (14.93%), *Apiotrichum* (3.14%), *unclassified_o_Saccharomycetales* (1.74%), and *Penicillium* (1.25%) (Figure 4D). At the OTU level, there were 47 shared OTUs among the three treatment groups, and the number of unique OTUs for the CK, LD, and HD samples were 164, 66, and 36, respectively (Figure S3).

### 2.6. Fungi Functional Prediction Analysis

The results of the functional predictions of fungal communities in *B. tabaci* are presented in Figure 4E. Compared to the relatively well-developed approaches for functional predictions in bacterial communities, the predictions for fungal communities based on ITS sequences are still in the early stage of development. At present, only a few broad categories could be predicted for fungal community functions. Here, among our samples, the “undefined saprotroph” category was the most frequent, followed by “soil saprotroph”, “animal pathogen”, and “unknown”. Among the three treatments, the HD group had the highest prevalence of the “undefined saprotroph” category. Interestingly, the LD group had a higher frequency of the “soil saprotroph” category than both the CK and the HD groups (Figure 4E).

## 3. Discussion

Overall, our study revealed that cadmium treatment had notable influences on both bacterial and fungal communities in whiteflies. For both the bacterial and fungal communities, the mean observed species richness (Sobs) decreased after cadmium treatment. However, the statistical significance of the changes differed between the bacterial and fungal communities. For the bacterial community, the decrease in Sobs was statistically not significant. In contrast, cadmium treatment significantly reduced the fungal Sobs. A similar pattern was observed for two other diversity indices, ACE and Chao1. In contrast, a reverse pattern was observed for the two remaining indices, where cadmium treatment caused significant increases in both the Shannon index and the Simpson’s index of diversity for the bacterial community but not for the fungal community in whiteflies.

Multiple indices have been proposed to describe microbial diversity, and most indices take into account both OTU richness and the relative abundances of individual OTUs. However, different indices place different weights on the observed OTU richness and the relative abundances of OTUs [25]. For example, both Shannon and Simpson’s indices estimate diversities by treating all OTUs equally and by considering both OTU richness and evenness, but with slight differences in their relative emphasis on either OTU richness (Shannon) or evenness among OTUs (Simpson). The observed increases in the Shannon and Simpson indices in the bacterial community in whiteflies after cadmium treatment suggest that the OTUs in the LD and HD treatments were overall more evenly distributed than in the CK treatment. In contrast, ACE and Chao1 estimate the expected species richness based on observed distributions of OTUs in each sample. Specifically, ACE estimates the expected species number by considering the relative frequencies of only OTUs with fewer than 10 individuals (i.e., <10 sequence reads in each sample in our study) [26]. For those with more than 10 individuals in the sample, only the presence or absence information is considered, while their exact frequencies are not considered in the ACE method. For the Chao1 OTU richness estimator, only the singletons and doubletons are used to estimate the predicted number of species in each sample [27]. Chao1 is frequently used for data sets skewed toward the low-abundance species. Our identification of a significantly higher number of expected fungal species in the CK samples than in the LD and HD samples, as indicated in the ACE and Chao1 indices, is also consistent with those observed for Sobs (Table 2). Indeed, the estimated fungal species richness by ACE and Chao1 were very close to those observed (Sobs) in all three treatment groups (Table 2). In contrast, on average, the estimated bacterial OTU numbers per sample based on ACE and Chao1 in all three treatment groups were all higher than those observed (Sobs) (Table 2). These results suggest additional undiscovered bacterial diversity in whiteflies in all three treatment groups.

In our study, Proteobacteria, Actinobateriota, Firmicutes, and Bacteroidota were the dominant bacterial phyla in all three treatment groups. Our results are similar to results obtained by Santos-Garcia et al., who demonstrated that Proteobacteria, Firmicutes, and Actinobacteria were the main bacterial groups of *B. tabaci* in pepper and watermelon [28]. Similarly, previous investigations reported that *B. tabaci* harbored *Candidatus_Portiera*, *Rickettsia*, *Candidatus_Hamiltonella*, *Arsenophonus*, *Cardinium*, and *Wolbachia* [21,22]. Bacteria of the genus *Candidatus_Portiera* are obligatory endosymbionts and have been reported in all *B. tabaci* analyzed so far [23,28]. Other symbiotic bacteria, for example, *Candidatus_Hamiltonella* and *Rickettsia*, are facultative endosymbionts [22]. It is worth noting that while bacterial genera such as *Rickettsia*, *Candidatus_Portiera*, and *Candidatus_Hamiltonella* have been previously reported in *B. tabaci*, our study reported for the first time that bacteria in the genera *Rhodococcus* and *Achromobacter* are also common members of the microbial community in *B. tabaci*.

Bacteria of the genus *Rhodococcus* are Gram-positive, aerobic, and have high G+C contents. They are non-motile and non-sporulating [29]. *Rhodococcus* can survive in hostile environments, such as those contaminated with polychlorinated biphenyl [30], nitrophenols, trichloroethene [31], and polycyclic aromatic hydrocarbons [32]. Studies have shown that *Rhodococcus* can degrade and remove aromatic [33], nitrile [34], sulfuric [35], and nitrogenous compounds [36] in the environment. Indeed, *Rhodococcus* are commonly used to remediate environmental contamination and pollution [37]. In our study, *Rhodococcus* was significantly enriched in *B. tabaci* in the HD compared to the control group. As previously reported, *Rhodococcus* strains are often resistant to cadmium [38]. Our results also suggest that *Rhodococcus* likely contribute to cadmium resistance in *B. tabaci*, and its increased abundance in the LD and HD groups was potentially related to the increased cadmium concentration in its diet.

In our study, the whiteflies used in the experiments were kept on pepper plants for over a year. We suspected that *Rhodococcus* in these whiteflies was obtained from the host plant *Capsicum* and these bacteria might have helped in reducing the toxicity of secondary metabolites such as capsaicin in pepper plants to these whiteflies. In pepper plants, the biosynthesis of capsaicin involves several substances containing a benzene ring or sulfur. We hypothesize that *Rhodococcus* could convert these substances to others and block the synthesis of capsaicin to reduce its toxicity to *B. tabaci*. Controlled experiments with axenic whiteflies and detailed chemical analyses of gut contents are needed to test this hypothesis. In addition to *Rhodococcus*, we also found an increased abundance of *Exiguobacterium* in cadmium-treated samples over that in the CK group. As previously reported, strains of *Exiguobacterium* exhibited an intrinsic resistance to cadmium [39] and could also play a role in reducing cadmium toxicity to whiteflies.

In our samples, *Rickettsia* was among the most abundant facultative endosymbionts in *B. tabaci.* Interestingly, *Rickettsia* could synthesize lysine due to the horizontal transfer of genes from *B. tabaci* [40]. In *B. tabaci*, *Rickettsia* has shown to play a positive role in improving the adult survival rate, egg production, the female adult ratio among offspring, high temperature tolerance, and the development rate [41,42]. We observed that cadmium treatment significantly reduced the relative abundance of *Rickettsia*, likely a contributor to the increased mortality of whiteflies in the presence of cadmium.

The PCoA of bacterial communities separated the CK and HD treatment groups. However, the PCoA of fungal communities failed to separate the three treatment groups into distinct clusters. Overall, the difference in bacterial community compositions among the three treatment groups was greater than that of fungal communities. The results suggested that cadmium treatments had an overall greater influence on bacterial communities than on fungal communities. The compositional differences in bacterial communities among the three treatment groups resulted in the increased prevalence of several predicted major metabolic pathways among the three treatments. Those pathways included “lipid metabolism”, “energy metabolism”, “xenobiotics biodegradation and metabolism”, and “metabolism of other amino acids”, consistent with cadmium having a significant influence on the metabolic pathways of whiteflies.

In all three treatments, *Wallemia* was the dominant fungal genus in *B. tabaci*. This Basidomycete genus contains several strongly xerophilic, xerotolerant, and halophilic species [43]. Thus far, *Wallemia* species have been primarily reported from indoor and outdoor air, dry feed for animals, hypersaline waters, and salted or highly sugared foods, but rarely from insects [43]. Importantly, the majority of *Wallemia* species can produce toxins and contaminate food [43]. In addition, three species in this genus (*W. sebi*, *W. muriae*, and *W. mellicola*) have been associated with human health problems, such as subcutaneous/cutaneous infections and farmer’s lung disease [43]. At present, while the role(s) of *Wallemia* in *B. tabaci* is unknown, the increased prevalence of *Wallemia* from ~57% to >70% upon cadmium exposure in this cosmopolitan agriculture pest suggests potentially significant risks that these fungi can pose to human health [44]. Further study is warranted to investigate the interactions between *Wallemia* and whiteflies and to understand the epidemiology and health impact of these fungi on crops and humans.

## 4. Materials and Methods

### 4.1. Insect Strain

*Bemisia tabaci* MED (Mediterranean) was originally obtained from a pepper plant (*Capsicum annuum* L.) field in Changsha, China, in 2020. These whiteflies were reared on pepper cultivar Zhongjiao 4 in an artificial climate box (L14: D10, 26 °C, 80% relative humidity (RH)). The quality of our experimental populations was maintained and monitored according to a previously published method [45].

### 4.2. Toxicity of Cadmium Exposure to B. tabaci

To develop the bioassay for testing cadmium toxicity for *B. tabaci* adults, whiteflies were directly fed the solutions containing cadmium for 48 h in a special feeding facility. The original feeding solution consisted of 30% sucrose and 5% yeast extract (wt/vol). This was the cadmium-free feeding solution and was used in the control (CK) treatment. The solutions with different cadmium concentrations (5, 15, 30, 60, and 120 µM) were prepared by dissolving anhydrous cadmium chloride (CdCl_2_, 99.99% pure, Sigma-Aldrich, St. Louis, MO, USA) into the original feeding solution. Each 200 µL feeding solution with different concentrations of cadmium was placed in each replicate sample of whiteflies in a designated feeding facility. There were four replications per treatment, and each replicate contained 60 adult whiteflies of mixed sexes. Finally, the treated *B. tabaci* adults were placed in an artificial climate chamber with a temperature of 26 °C, 80% RH, and a photoperiod of L14:D10. The toxicity of each cadmium concentration to *B. tabaci* was estimated based on the death rate of *B. tabaci* at 48 h after cadmium exposure.

### 4.3. Sample Preparation

According to the results of cadmium toxicity for *B. tabaci*, the final concentrations of 0 (control, marked as CK), LC10 (the concentration of cadmium exposure that caused 10% death rate of whiteflies, at 0.186 mg/kg in artificial diet marked as LD), and LC50 (the concentration of cadmium exposure that caused 50% death rate of whiteflies, at 1.71 mg/kg in artificial diet, marked as HD) were selected for microbial diversity assessment. About 120 *B. tabaci* adults were included in each of the three treatments (CK, LD, and HD). Each treatment was performed four times. After 48 h, surviving whiteflies were collected, washed with 70% alcohol twice, rinsed with sterile water three times, flash-frozen in liquid nitrogen, and stored in a −80 °C freezer until their microbial diversities were investigated.

### 4.4. Amplicon Sequencing

Total DNA was extracted from the *B. tabaci* adults using the E.Z.N.A.^®^ DNA Kit (Omega Bio-tek, Norcross, GA, USA) following the operating instruction’s guidance. The primers for the bacterial DNA amplification were 338F (5′-ACTCCTACGGGAGGCAGCAG-3′) and 806R (5′-GGACTACHVGGGTWTCTAAT-3′). The primers for the fungal DNA amplification were ITS1F (5′-CTTGGTCATTTAGAGGAAGTAA-3′) and ITS2R (5′-GCTGCGTTCTTCATCGATGC-3′). The PCR amplification, the Illumina MiSeq sequencing, and the analysis of sequencing data were performed following the procedures described in our previous study [14]. The raw reads have been deposited in the NCBI Sequence Read Archive (SRA) database (BioProject Number: PRJNA957946; sequence accession numbers SRR24235724-SRR24235735).

### 4.5. Statistical Analysis

The alpha diversity indices were measured by Optifit and the beta diversity indices were measured by Bray–Curtis distance matrices [46]. All the data that needed to be analyzed statistically were tested by one-way analysis of variance (ANOVA) with LSD’s range test using SPSS (the Statistical Package for the Social Sciences, version 20.0, Chicago, IL, USA); *p* values < 0.05 were considered statistically significant. The diversity values were expressed as the mean ± standard error. Principal co-ordinate analysis (PCoA) and hierarchical clustering of bacterial and fungal communities were conducted based on the Bray–Curtis distance among samples. Heatmaps were generated using Heml (version 1.0). Bacterial and fungal functional groups in *B. tabaci* under different concentrations of cadmium were predicted by PICRUSt2 [47] and FUNGuild [48].

## 5. Conclusions

In our study, the influences of cadmium on the bacterial and fungal communities of whiteflies were investigated. Cadmium exposure reduced fungal community alpha diversity but increased the bacterial community Shannon and Simpson’s diversity indices. We found that *Rhodococcus* and *Achromobacter* were among the dominant genera of the bacterial community in *B. tabaci*. Compared with the control, the HD treatment showed a significant enrichment of OTUs in the genera *Rhodococcus* and *Exiguobacterium*, consistent with their potential roles in the resistance to cadmium and in reducing cadmium toxicity to whiteflies. In addition, cadmium treatment increased the relative abundance of bacterial taxa involved in “lipid metabolism”, “xenobiotics biodegradation and metabolism”, and “energy metabolism” in *B. tabaci*. Of special interest is the high and increased abundance of the fungal genus *Wallemia* upon exposure to cadmium in whiteflies. The high relative abundances of *Rhodococcus* and *Wallemia* in our samples suggested potentially essential roles of these microbes for *B. tabaci* in China. Our results also suggested potential novel strategies for controlling *B. tabaci* by the targeted disturbance of *Rhodococcus* and/or *Wallemia* using chemical and/or biological agents to reduce their contributions to the survival and reproduction of whiteflies and consequently to limit the damage from the whitefly pests on crops. Furthermore, understanding the mechanisms of how *Rhodococcus* and/or *Wallemia* reduce cadmium toxicity in whiteflies could also help us develop strategies to minimize the negative effects of cadmium contamination in agricultural fields and improve food security and human health.

## Figures and Tables

**Table 1:** Death rates of *B. tabaci* at different cadmium concentrations.

Concentration of Cadmium (μmol/L)	Number of Tested Insects	Death Rate
120	252	74.6%
60	260	65.00%
30	208	41.35%
15	261	26.05%
5	264	14.02%
0	263	12.93%

**Table 2:** Diversity indices (mean ± standard error, *n* = 4) of bacterial and fungal communities in *B. tabaci* treated with different concentrations of cadmium.

Diversity Indices *		Treatment		
		CK	LD	HD
Bacterial community	Sobs	90.75 ± 39.45 a	55.75 ± 24.58 a	79.75 ± 30.31 a
	Shannon	0.78 ± 0.05 b	0.84 ± 0.02 ab	0.99 ± 0.07 a
	Simpson	0.35 ± 0.01 a	0.43 ± 0.02 b	0.48 ± 0.03 b
	ACE	186.09 ± 41.05 a	112.47 ± 24.03 a	102.16 ± 37.04 a
	Chao1	128.09 ± 46.26 a	74.32 ± 28.68 a	97.04 ± 35.09 a
Fungal community	Sobs	97.25 ± 7.03 a	57.25 ± 7.35 b	37.50 ± 6.61 b
	Shannon	1.72 ± 0.17 a	1.31 ± 0.20 a	1.07 ± 0.26 a
	Simpson	0.60 ± 0.08 a	0.48 ± 0.07 a	0.42 ± 0.12 a
	ACE	98.88 ± 5.99 a	60.36 ± 5.94 b	37.73 ± 6.68 c
	Chao1	98.20 ± 6.42 a	58.75 ± 6.88 b	37.5 ± 6.61 b

*, Sobs: sum of observed number of species; Shannon: Shannon–Weaver diversity index; Simpson: Simpson’s index of diversity 1-D; ACE: abundance-based coverage estimator of expected species richness; Chao1: abundance-based estimator of expected species richness. Different letters (a, b, or c) in the same row indicate significant differences (*p* < 0.05).

## Data Availability

All microbiome data reported here have been deposited in GenBank.

## Supplementary Materials

The following supporting information can be downloaded at: https://www.mdpi.com/article/10.3390/ijms241713588/s1.
