# Supplementary material for: Effects of Cadmium Stress on Bacterial and Fungal Communities in the Whitefly Bemisia tabaci
Source: Int J Mol Sci. 2023 Sep 2;24(17):13588. doi: 10.3390/ijms241713588 (PMC10488276; doi:10.3390/ijms241713588)
Supplement: Supplementary file 1 [file ijms-24-13588-s001.zip › Supplementary files/Supplementary Materials-Whitefly microbiome.pdf]

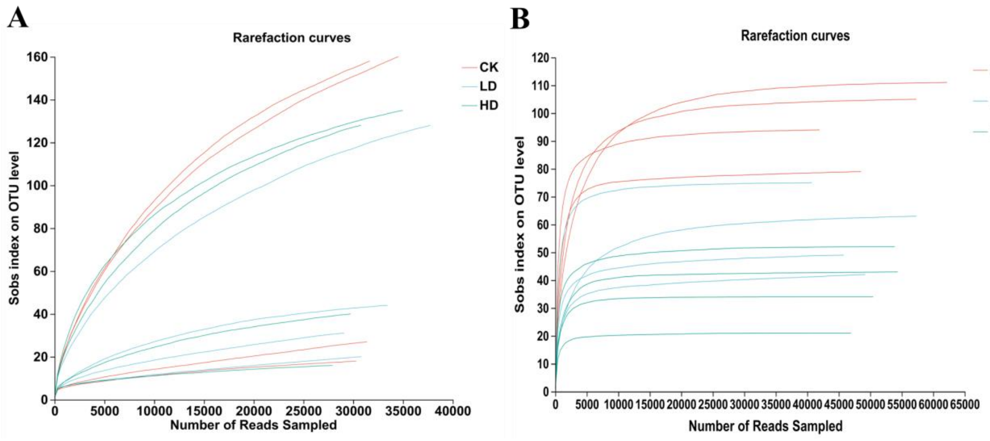

Figure S1. Rarefaction curves of OTUs clustered at 97% sequence identity with the 16S rRNA and ITS sequences from *B. tabaci* treated with different concentrations of cadmium. A Rarefaction curves of bacterial OTUs based on 16S rRNA sequence reads. **B** Rarefaction curves of fungal OTUs based on ITS sequence reads. Sobs represents the observed number of species/OTUs.

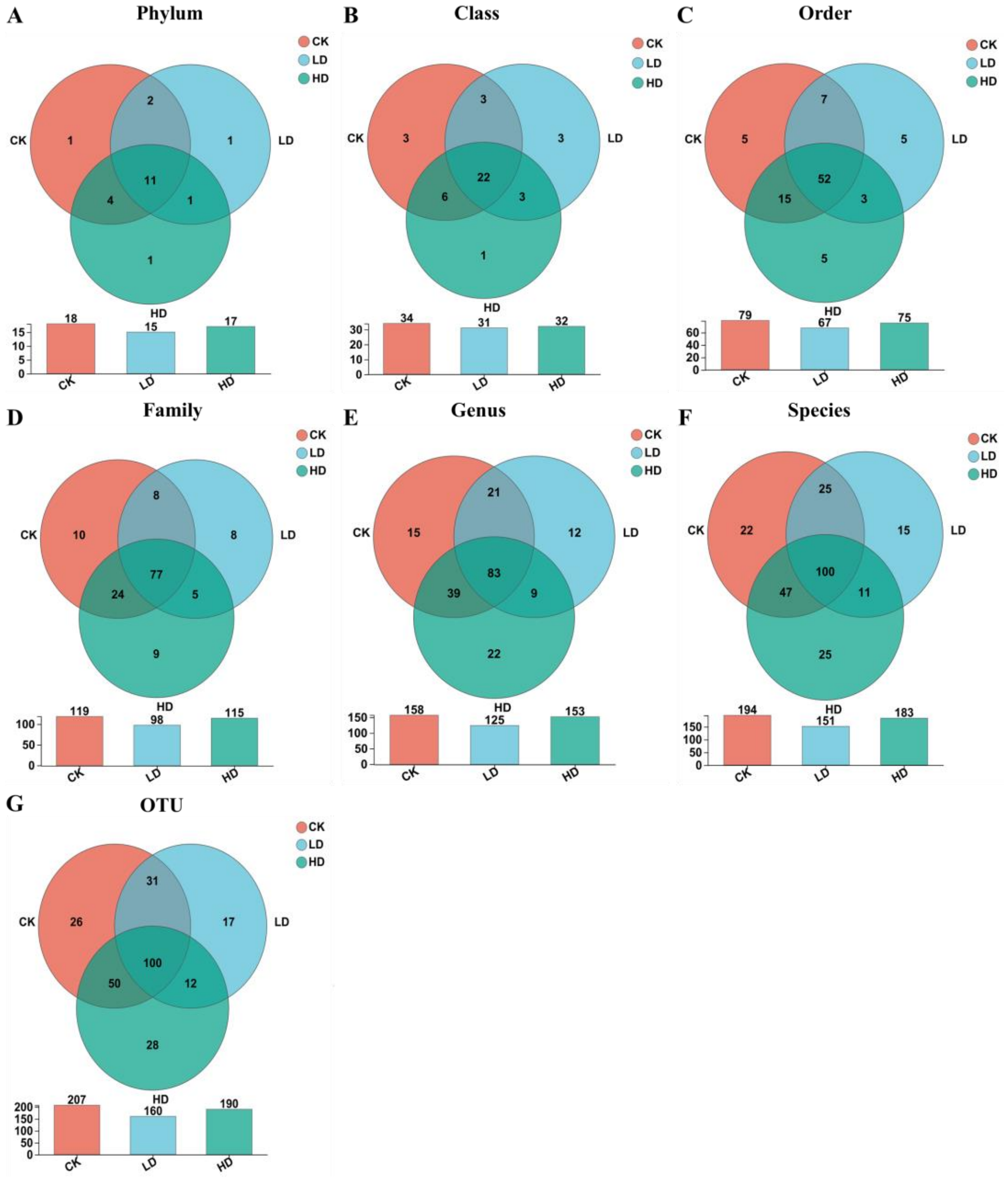

Figure **S2. Venn diagram among bacterial communities in *B. tabaci* treated with different concentrations of cadmium.** The number in the non-overlapping region represented endemic species number to each treatment and common species number to all treatments in the middle. Below each venn diagram is a histogram of the number of total bacteria in each treatment.

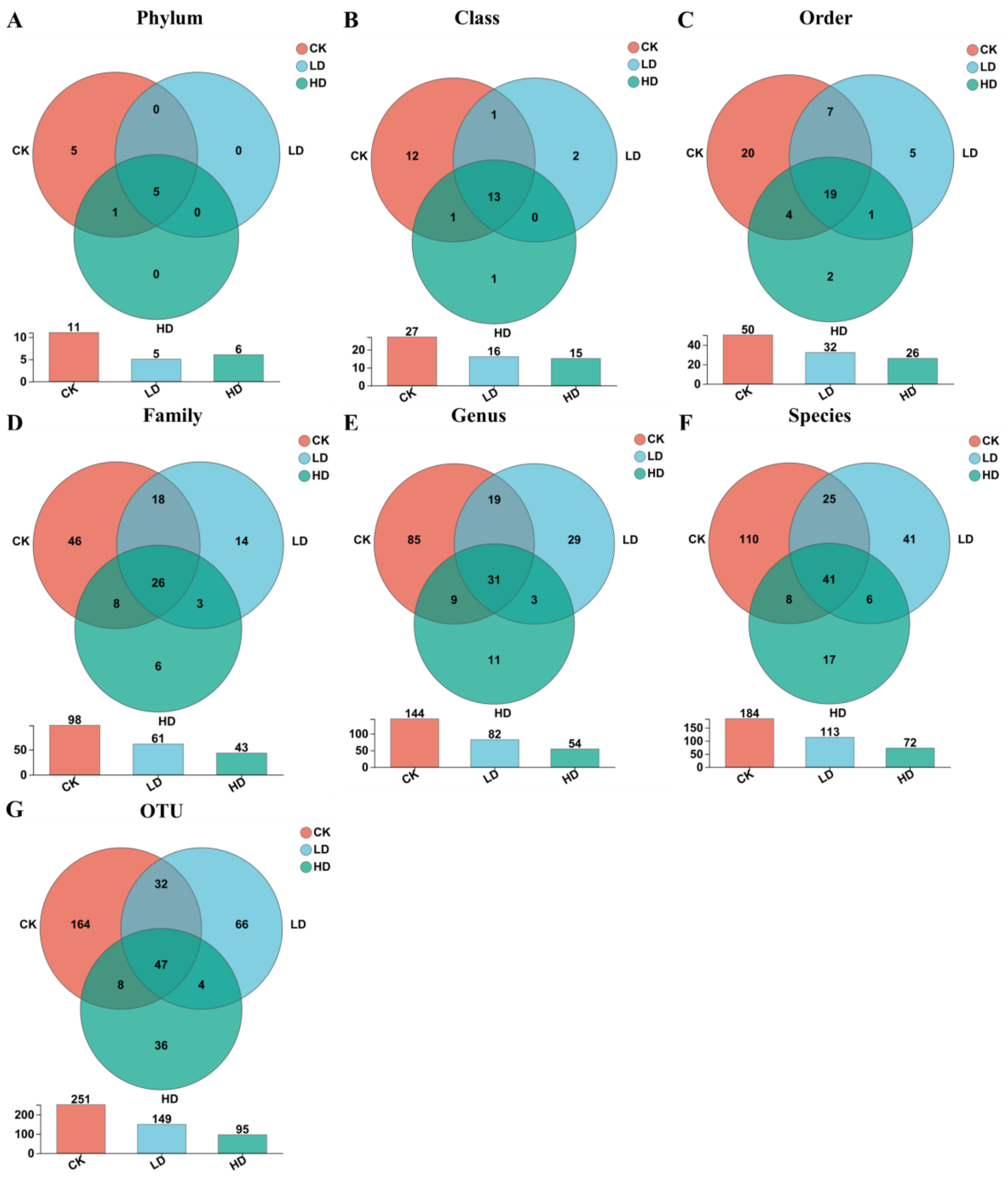

Figure S3 Overlap in fungal community composition at different taxonomic levels in *B. tabaci* treated with different concentrations of cadmium. The number in the non-overlapping region represented endemic species number to each treatment and common species number to all treatments in the middle. Below each venn diagram is a histogram of the number of total fungi in each treatment.

Table S1 The list of bacterial OTUs based on 16S rRNA sequences in *B. tabaci* treated with different concentrations of cadmium.

Table S2 The list of fungal OTUs based on ITS sequences in *B. tabaci* treated with different concentrations of cadmium.

Table S3 Summary of the 16S rRNA and ITS read counts of bacteria and fungi in *B. tabaci* treated with different concentrations of cadmium.

**Summary of read counts**		**Sample**		
		**CK**	**LD**	**HD**
**Bacteria**	**Read Number**	34579.00±1132.46a	34011.00±1592.10a	31866.00±1349.28a
	**Mean length (bp)**	406.86±0.21b	407.08±0.11b	407.90±0.33a
	**Phylum**	9.50±2.96a	8.25±1.18a	9.75±1.97a
	**Class**	18.00±5.96a	14.50±3.66a	17.50±3.93a
	**Order**	42.00±14.91a	28.75±8.43a	40.00±11.45a
	**Family**	60.25±23.02a	39.25±14.02a	55.00±17.84a
	**Genus**	72.50±29.52a	45.75±18.17a	66.50±23.56a
	**Species**	86.25±36.85a	53.25±23.39a	78.00±29.62a
	**OTU Number**	90.75±39.45a	55.75±24.58a	79.75±30.31a
**Fungi**	**Read Number**	52572.75±4490.27a	48271.25±3486.07a	51441.75±1728.02a
	**Mean length (bp)**	190.56±6.62a	195.34±5.06a	185.16±4.60a
	**Phylum**	6.25±0.75a	3.50±0.50b	4.00±0.41b
	**Class**	16.50±1.71a	10.25±0.63b	10.00±0.71b
	**Order**	29.00±2.04a	16.75±2.06b	13.75±0.85b
	**Family**	49.00±2.80a	26.00±4.56b	18.25±2.21b
	**Genus**	61.75±3.42a	33.50±5.95b	22.50±3.01b
	**Species**	80.00±5.11a	47.50±5.91b	30.00±4.38c
	**OTU Number**	97.25±7.03a	57.25±7.35b	37.50±6.61b

Given data are the mean ±standard errors of four replicates. Different letters in the same line indicate significant differences ($P<0.05$).
